# Role of metastasis-associated protein 1 in prognosis of patients with digestive tract cancers: A meta-analysis

**DOI:** 10.1371/journal.pone.0176431

**Published:** 2017-06-01

**Authors:** Guo-dong Cao, Bo Chen, Mao-ming Xiong

**Affiliations:** 1 Anhui Medical University, Hefei, Anhui, China; 2 Department of General Surgery, The First Affiliated Hospital of Anhui Medical University, Hefei, Anhui, China; Hospital Universitario de Albacete, SPAIN

## Abstract

**Objectives:**

Metastasis-associated protein 1 **(**MTA1) is a transcriptional regulator and significantly associated with prognosis of patients with cancer. However, its role as a potential prognostic marker in digestive tract cancer (DTC) is controversial. In this study, a meta-analysis was conducted to evaluate the MTA1 expression as a predictor of clinicopathology and survival of patients with DTC.

**Methods:**

We searched PubMed, Ovid, Web of Science and Cochrane databases using multiple search strategies for eligible studies. STATA 11.0 software was used to pool the data and analyze the association, odds ratios (ORs) and 95% confidence intervals (CIs) were used to measure the strength of the association. Furthermore, the Newcastle-Ottawa scale was used to evaluate the quality of eligible studies.

**Results:**

MTA1 overexpression was strongly associated with depth of invasion (OR = 1.88, 95%CI: 1.05–3.37, *P* = 0.03), lymph node metastasis (OR = 2.30, 95%CI: 1.76–3.01, *P*<0.001), vascular invasion (OR = 2.02, 95%CI: 1.40–2.91, *P*<0.001) and TNM stage (OR = 2.78, 95%CI: 1.63–4.74, *P*<0.001), and was related to 1- (RR = 1.84, 95%CI: 1.18–2.89, *P* = 0.008), 3- (RR = 1.74, 95%CI: 1.32–2.30, *P<*0.001) and 5-year (RR = 1.64, 95%CI: 1.18–2.27, *P* = 0.003) OS. Further, MTA1 was associated with 1- (RR = 4.16, 95%CI: 1.35–12.81, *P* = 0.01), 3- (RR = 1.90, 95%CI: 1.02–3.53, *P* = 0.04) and 5- (RR = 2.17, 95%CI: 1.41–3.32, *P*<0.001) year DFS. In subgroup analyses based on study quality and tumor type, MTA1 overexpression was obviously related to clinical parameters, such as lymph node metastasis and TNM stage, and was also associated with prognosis of patients with gastrointestinal or esophageal cancer.

**Conclusions:**

MTA1 expression is strongly correlated with metastasis-related variables, and represents a promising prognostic factor in DTC.

## Introduction

Digestive tract cancers (DTCs) are a heterogeneous group of gastrointestinal (GI) cancers as well as hepatobiliary and pancreatic tumors. DTCs are important causes of cancer-related deaths worldwide [1–2]. Data from the Global Cancer Statistics, 2012 [1] indicate that colorectal cancer (CRC), gastric cancer (GC), and esophageal cancer (EC) rank fourth, sixth, and tenth among all DTCs.

DTC increases the risk of lymph node metastasis and distant metastasis. Lymph node status and distant metastasis are included in tumor staging, which is the most useful indicator in predicting outcomes. However, adequate evidence reminds us of the inability of conventional staging criteria to differentiate prognostic features of DTC. Metastasis is a multi-step process encompassing dissemination of primary cancer cells and subsequent colonization at distant sites [3], and is the overwhelming cause of morbidity and mortality inpatients with cancer. Therefore, elucidation of the mechanism and development of new strategies to prevent metastasis are essential to combating cancers.

Several factors are associated with the prognosis of cancer and regulation of metastasis. Metastasis-associated proteins (MTAs), especially MTA1, play prominent roles.MTA1 belongs to MTA family (consisting of MTA1, MTA2 and MTA3) that is associated with the nucleosome remodeling and histone deacetylation (NuRD) complex, which regulates transcription via histonedeacetylation and chromatin remodeling [4]. MTA1 was first reported in metastatic rat breast adenocarcinoma cell lines, where it was highly expressed compared with poorly metastatic cell lines. It plays a key role as a tumor invasion and metastasis-related gene [5]. Toh *et al*. [6] found that higher mRNA levels of MTA1 were closely related to depth of invasion and lymph node metastasis and a tendency toward a higher rate of lymphatic involvement. Song *et al*. [7] found that overexpression of MTA1protein is an independent prognostic risk factor, and is associated with shorter disease-free survival and lower 5-year survival rate.

To the best of our knowledge, only one meta-analysis reviewed the prognostic significance of MTA1 in solid tumors. The prognostic value of MTA1 in DTC is inconclusive and controversial. Therefore, we conducted a meta-analysis to investigate the role of MTA1 expression in the prognosis and survival of patients with DTC.

## Methods

### Search strategy

Two investigators independently searched PubMed, Ovid, Web of Science, Cochrane databases for studies published until Jul 2016. The search terms used were:("MTA1" OR "Metastasis-associated protein 1") AND ("esophagus" OR "esophageal" OR "oesophagus" OR "gullet" OR "esophago-cardiac" OR "colon" OR "colorectal" OR "rectal" OR "anal" OR "pancreas" OR "pancreatic" OR "liver" OR "hepatic" OR " biliary duct" OR "bile duct" OR "gastric" OR "stomach" OR "cardia" OR "digestive tract") AND ("carcinoma" OR "cancer" OR "tumour" OR "neoplasm" OR "tumor" OR"malignancy"). The full texts of the studies were retrieved to determine their eligibility for inclusion in the meta-analysis.

### Inclusion and exclusion criteria

The inclusion criteria were: (1) DTC diagnosis; (2)studies using immunohistochemistry (IHC); (3) correlation between MTA1 and DTC; and (4) studies published in English language. The exclusion criteria were: (1)redundant data; (2) reviews; (3) case reports; (4) studies without IHC analysis; and (5) inaccurate data.

### Data extraction and assessment

All the pertinent data were extracted independently from each eligible study by two investigators (Guo-dong Cao, Bo Chen). Any disagreement was resolved through discussion until a consensus was reached. The following data were extracted: first author’s name, year of publication, total number of patients, clinicopathological parameters, and survival time. Two researchers independently evaluated the quality of eligible studies using the Newcastle- Ottawa scale [8].

### Statistical analysis

All the statistical analyses were performed using the STATA software (version 11.0, StataCorp LP, College Station, TX, USA).The crude odds ratios (OR) and95% confidence intervals (CI) were used to estimate the strength of association between MTA1 and clinicopathological parameters. Risk ratios (RR) and 95% CIs were used to estimate the association of MTA1 status with the overall survival (OS) and disease-free survival (DFS). *I*^2^ value, which indicated the percentage of total variation across studies, was used to assess statistical heterogeneity. Random-effects models (*I*^2^>50% or *P*<0.10) were used if significant heterogeneity was detected. Otherwise, fixed-effects models were used. Begg's rank correlation and Egger's weighted regression were used to determine potential publication bias. *P* value less than0.05 indicates statistically significant publication bias.

## Results

### Study characteristics

**Fig 1:**
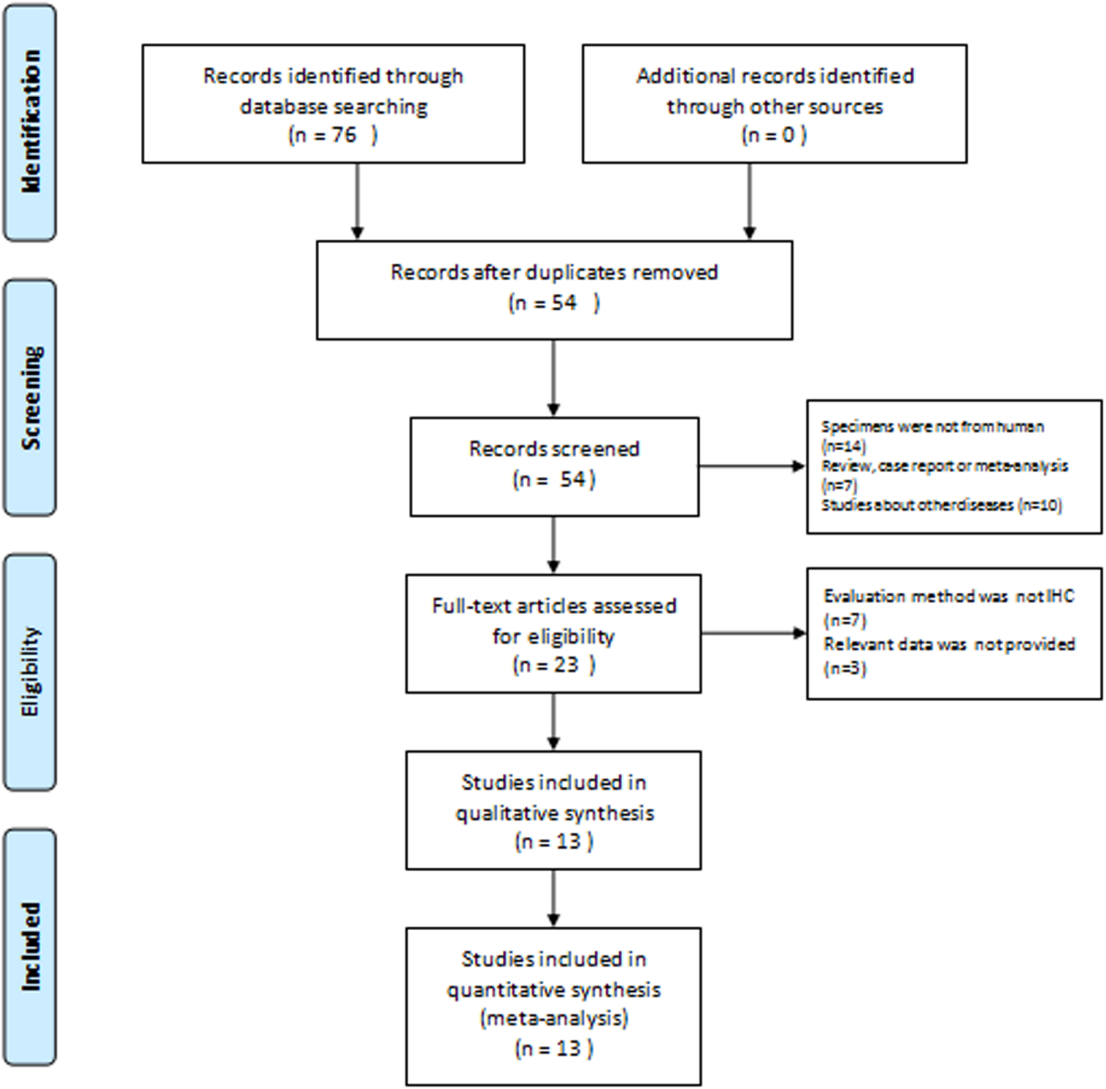
Flow diagram of study selection procedure.

The search strategy identified 76 studies potentially eligible for the relationship betweenMTA1 protein overexpression and DTC. After reading the titles, 23 studies were probably eligible. After browsing the abstracts and full text, 5 studies on MTA1 and EC [7, 9–12], 3 studies on MTA and GC [13–15], 2 studies on MTA1 and CRC [16–17], 2 studies on MTA1 and liver cancer [18–19] and one study on MTA1 and pancreatic cancer [20] met the inclusion criteria, respectively (Fig 1). Full details of all the included studies are summarized in Table 1 and Table 2. TheMTA1 expression in 1,997 DTC patients was studied, and the number of patients ranged from 39 to 506 patients in 13 different included studies. These studies used immunohistochemistry (IHC) to analyze the MTA1 status of DTC samples, and results were performed in their studies. Furthermore, the overall MTA1 positive expression rate in DTC patients was 35.8% (714/1997). Over-expression rate was 43.7% (289/662) in EC, 47.3% (157/332) in GC, 40.6% (63/155) in CRC, 33.3% (13/39) in pancreatic cancer and 23.7% (192/809) in liver cancer, respectively.

**Table 1:** Clinicopathological parameters and quality scores of studies comparingMTA1 positive digestive tract cancer with MTA1 negative digestive tract cancer.

Study	Year	Tumor type	Number of patient	Sex		Age		Tumor size		Differentiation		Depth of invasion		LN metastasis		Distant metastasis		Tumor stage		Vascular invasion		Quality score
				male	female	<60	>60	<5cm	>5cm	well	poor	T1+T2	T3+T4	positive	negative	positive	negative	early	advanced	positive	negative	
				MTA1(+)	MTA1(-)	MTA1(+)	MTA1(-)	MTA1(+)	MTA1(-)	MTA1(+)	MTA1(-)	MTA1(+)	MTA1(-)	MTA1(+)	MTA1(-)	MTA1(+)	MTA1(-)	MTA1(+)	MTA1(-)	MTA1(+)	MTA1(-)	
Toh	2004	Esophageal	70(30vs.40)	24/6	37/3	NA		NA		22/8	26/14	10/20	30/10	21/9	15/25	NA		13/17	28/12	15/15	11/29	9
Yang	2016	Esophageal	197(83vs.114)	63/20	85/29	38/45	55/59	NA		65/19	93/21	28/55	58/56	45/38	43/71	NA		NA		NA		8
Li	2012	Esophageal	131(57vs.74)	40/17	55/19	NA		36/21	57/17	45/12	50/24	18/39	38/36	37/20	33/41	NA		26/31	55/19	NA		8
Li	2009	Esophageal	90(40vs.50)	28/12	34/16	NA		24/16	35/15	30/10	31/19	11/29	27/23	NA		NA		NA		NA		8
Song	2013	Esophageal	174(79vs.95)	60/19	70/25	NA		48/31	63/32	63/16	76/19	18/61	41/54	48/31	43/52	NA		41/38	64/31	NA		5
Deng	2013	Gastric	111(40vs.71)	30/10	57/14	NA		23/17	67/4	17/23	43/28	31/9	58/13	NA		NA		NA		NA		6
Yao	2015	Gastric	61(47vs.14)	16/31	7/7	NA		NA		NA		NA		37/10	6/8	NA		7/40	10/4	NA		8
Meng	2015	Gastric	160(70vs.90)	47/23	67/23	NA		47/23	62/28	NA		14/56	35/55	59/11	58/32	NA		NA		31/39	26/64	8
Higashijima	2011	Colorectal	74(38vs.36)	25/13	20/16	NA		NA		37/1	33/3	14/24	21/15	22/16	17/19	15/23	12/24	12/26	12/24	21/17	12/24	6
Du	2011	Colorectal	81(25vs.56)	17/8	28/28	11/14	23/33	10/15	36/20	15/10	44/12	3/22	12/44	17/8	23/33	NA		8/17	33/23	NA		8
Miyake	2007	Pancreatic	39(13vs.26)	8/5	18/8	NA		NA		11/2	26/0	NA		9/3	17/9	2/11	3/23	NA		NA		6
Jin	2012	Hepatic	303(104vs.199)	74/30	167/32	NA		72/32	123/76	NA		NA		NA		NA		NA		26/78	32/167	7
Ryu	2008	Hepatic	506(88vs.418)	NA		NA		NA		NA		NA		NA		NA		NA		NA		7

LN: lymph node; NA: not available

TNM stages are based on tumor-node-metastasis classification advocated by International Union against Cancer

Quality score: use the Newcastle-Ottawa scale (stars)

**Table 2:** IHC antibodies and assessment methods of MTA1 expression in the eligible studies.

Study	Year	Tumor Type	Antibody	Antibody Concentration	The Positive-cell Scoring	Staining Intensity	IHC Assessment Method
Toh	2004	Esophageal	NA	NA	NA	Score: no staining (0); slight staining (+); moderate staining (++); intense staining (+++).	Scores were compared between the carcinoma tissues and the normal squamous epithelium contained in the same section. In all cases, the normal epithelial cells were scored (+), and the scores (++) and (+++) in the carcinoma tissues were defined as overexpression of MTA1 protein
Yang	2016	Esophageal	sc-9446, Santa Cruz Biochemistry	1/100	Positive: <5%, 0 points; 5–25%, 1 point; 26–50%, 2 points; 51–75%, 3 points; and >75%, 4 points.	Staining intensity: Minimal staining similar to the background, 0 points; lightly stained, more than the background and pale yellow, 1 point; moderately stained, markedly more than the background and a brown-yellow, 2 points; and clearly stained a dark brown-yellow or tan, 3 points.	Total score: number of positive cells x staining intensity. Total score ≥5 indicated a positive result, and <5 indicated a negative result.
Li	2012	Esophageal	sc-9446, Santa Cruz Biotechnology	1/100	Positive: 0, 0–5%; 1,6–25%; 2, 26–50%; 3, 51–75%; 4, >76%	Staining intensity: 0, negative; 1, weak; 2, moderate; 3,strong	The final staining score was the sum of the scores of staining intensity and percentage of positive cells, ranging from 0 to 7.
Li	2009	Esophageal	sc-9446, Santa Cruz Biotechnology	1/100	NA	Staining intensity and proportion of the stained tumor nuclei as follows: score 0, no staining; (+), slight staining; (++), moderate staining;(+++), intense staining.	For all cases, the normal epithelial cells that were scored (+), and the cancer tissues that were scored (++) and (+++) were defied as overexpression of MTA1 protein
Song	2013	Esophageal	sc-9446, Santa Cruz Biochemistry	1/100	Positive: 0, 0–5%; 1, 6–25%; 2, 26–50%; 3, 51–75%; 4, >76%	Staining intensity: 0, negative staining; 1, weak staining; 2,moderate staining; 3, intense staining	The final staining score was the sum of the scores of staining intensity and percentage of positive cells: (-), 0 to 1; (+), 2 to 3;(++), 4 to 5; (+++), 6 to 7.
Deng	2013	Gastric	sc-9446, Santa Cruz Biochemistry	1/100	NA	The results were reported as follows: 0, no staining; +, slight staining; ++, moderate staining; +++, intense staining.	The cancer tissues scored as ++ and +++ were defined as exhibiting overexpression of MTA1 protein.
Yao	2015	Gastric	Santa Cruz Biochemistry	1/500	Positive: 0%, negative, 5%, weak positive; 5%–25%, intermediate; 25%–50%, moderate; 50%–100%, strong)	NA	The distribution of tumor cells in all experimental groups was determined as follows: 0%–5%is lower expression and 5%–100% is higher expression.
Meng	2015	Gastric	# 5647, Cell Signaling	1/100	<25%, 1; 25–50%, 2; >50%-<75%, 3; >75%, 4 scores	Staining intensity: negative, 0; weak, 1; moderate, 2; or strong, 3 scores	A staining index (values 0–12), >6 indicated a positive result.
Higashijima	2011	Colorectal	sc-17773, Santa Cruz Biochemistry	1/10	NA	NA	Regarding the assessment of staining, the tumor was defined as exhibiting positive staining when >10% nuclear staining of the protein was noted in the tumor tissue.
Du	2011	Colorectal	sc-9446, Santa Cruz Biochemistry	NA	Samples with 10% tumor cells were defined as positive.	Staining intensity: 0 (no staining), 1 (weak staining), 2 (moderate staining), and 3 (strong staining)	Tumors with a score > 2 (moderate and strong expression) showed a high expression level of MTA1.
Miyake	2007	Pancreatic	sc-17773, Santa Cruz Biochemistry	1/5	Samples with staining 10% of the tumor cells were defined as positive.	Staining intensity:: negative (score = 0), weak (score = 1), moderate (score = 2), or strong (score = 3)	Tumors with scores of more than 2 (moderate and strong expression) were considered to show MTA1 overexpression.
Jin	2012	Hepatic	NA	1/150	NA	NA	MTA-1 overexpression was defined when at least a portion of tumor cells (>5%) showed a positive MTA-1 staining.
Ryu	2008	Hepatic	Santa Cruz Biochemistry	1/200	NA	NA	(1) 0% (none, -); (2) MTA1 low group (less than 50%, +); and (3) MTA1 high group (more than 50%, ++).

### Relationship between MTA1 expression and clinicopathological parameters

**Fig 2:**
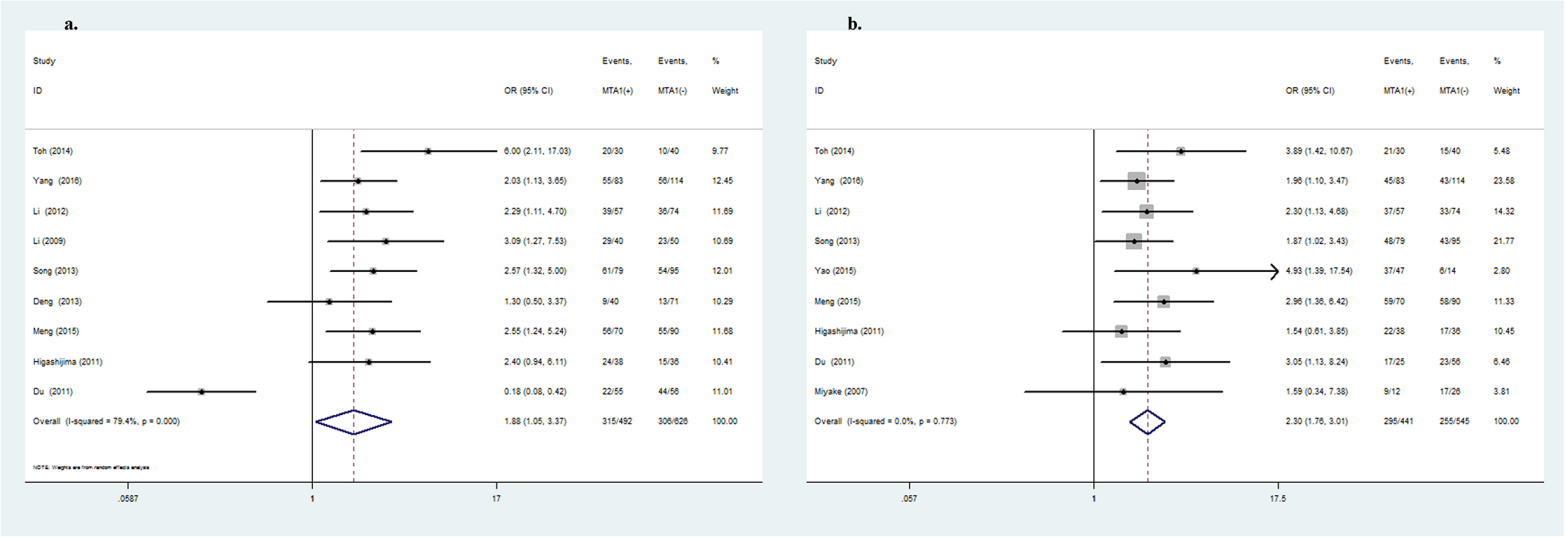
Forrest plot of odds ratio for the association of MTA1 and clinicopathlogical parameters. (2a) Association between MTA1 expression and depth of invasion. (2b) Association between MTA1 expression and lymph node metastasis.

**Fig 3:**
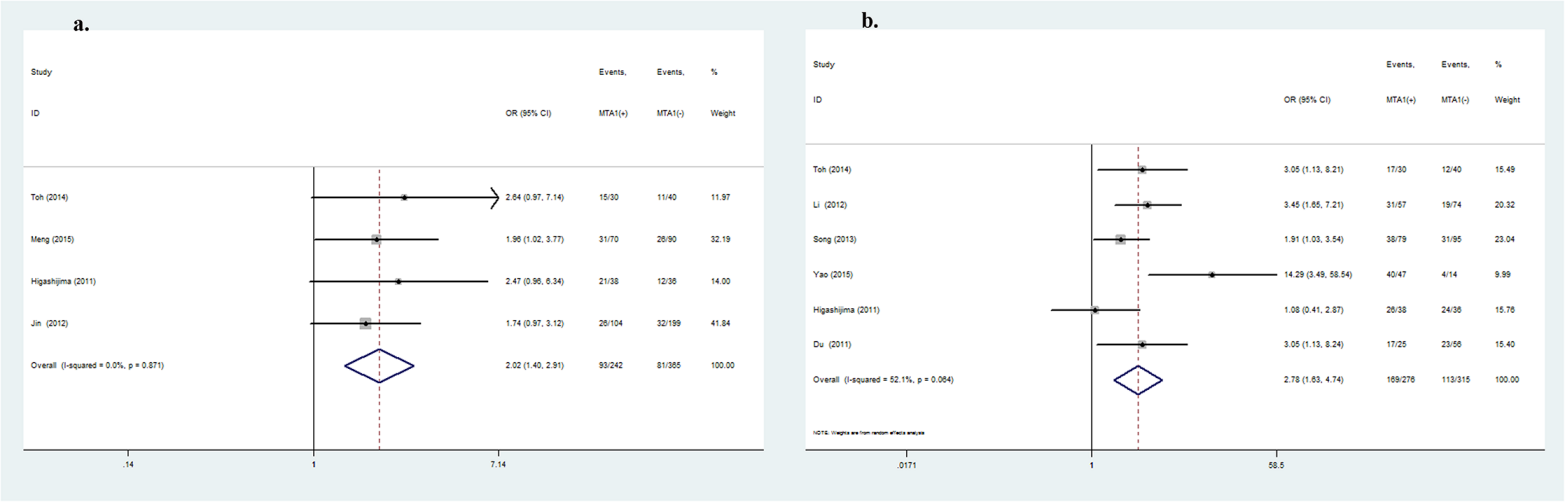
Forrest plot of odds ratio for the association of MTA1 and clinicopathlogical parameters. (3a) Association between MTA1 expression and vascular invasion. (3b) Association between MTA1 expression and TNM stage.

MTA1-positive expression was significantly associated with several types of metastasis-related clinical parameters. As shown in Table 3, MTA1 over-expression was strongly correlated with depth of invasion (OR = 1.88, 95%CI: 1.05–3.37, *P* = 0.03, Fig 2A), lymph node metastasis (OR = 2.30, 95%CI: 1.76–3.01, *P*<0.001, Fig 2B), vascular invasion (OR = 2.02, 95%CI: 1.40–2.91, *P*<0.001, Fig 3A) and TNM stage (OR = 2.78, 95%CI: 1.63–4.74, *P*<0.001, Fig 3B). MTA1-positive expression increased the risk for stomach wall invasion, lymph node-positive metastasis and vascular invasion, leading to a later TNM stage. Other clinicopathological variables such as gender, age, tumor size, differentiation or distant metastasis were not correlated with MTA1 expression.

**Table 3:** Meta-analysis of a putative association between clinicopathological parameters and MTA1 expression in digestive tract cancer.

Parameters	Number of studies	Number of patients	Heterogeneity		Model	OR(95%CI)	*P* value
			*I*^2^(%)	*P* value			
Sex (male/female)	12	1491	16	0.29	FE	0.84(0.67,1.07)	0.16
Age (<60/>60)	2	278	0	0.70	FE	0.96(0.59,1.56)	0.87
Tumor size (<5cm/>5cm)	7	1050	74	0.001	RE	0.58(0.34,1.01)	0.06
Differentiation (well/poor)	9	968	44	0.07	FE	1.06(0.78,1.43)	0.71
Depth of invasion (T1+T2/T3+T4)	9	1118	79	0	RE	1.88(1.05,3.37)	**0.03**
LN metastasis (positive/negative)	9	986	0	0.77	FE	2.30(1.76,3.01)	**<0.001**
Metastasis (positive/negative)	2	113	0	0.95	FE	1.32(0.56,3.10)	0.52
Tumor stage (early/advanced)	6	591	52	0.06	RE	2.78(1.63,4,74)	**<0.001**
Vascular invasion(positive/negative)	4	607	0	0.87	FE	2.02(1.40,2.91)	**<0.001**

LN metastasis: lymph node metastasis

TNM stages are based on tumor-node-metastasis classification advocated by International Union against Cancer

OR: odds ratio; CI: confidence interval; FE: fixed-effect model; RE: random-effect model

### Correlation of MTA1 overexpression with OS and DFS

Survival time was extracted from Kaplan–Meier survival curves analyzed by the Enguage Digitizer software. In the present study, as shown in Table 4, MTA1 expression was not only clearly linked to OS, but also showed significant association with DFS. DTC patients with MTA1-positive expression manifest shorter OS. MTA1 expression was significantly correlated with 1- (RR = 1.84, 95%CI: 1.18–2.89, *P* = 0.008), 3- (RR = 1.74, 95%CI: 1.32–2.30, *P*<0.001) and 5- (RR = 1.64, 95%CI: 1.18–2.27, *P* = 0.003, Fig 4A) year OS. Further, MTA1 expression was linked to1- (RR = 4.16, 95%CI: 1.35–12.81, *P* = 0.01), 3- (RR = 1.90, 95%CI: 1.02–3.53, *P* = 0.04) and 5- (RR = 2.17, 95%CI: 1.41–3.32, *P*<0.001, Fig 4B) year DFS.

**Fig 4:**
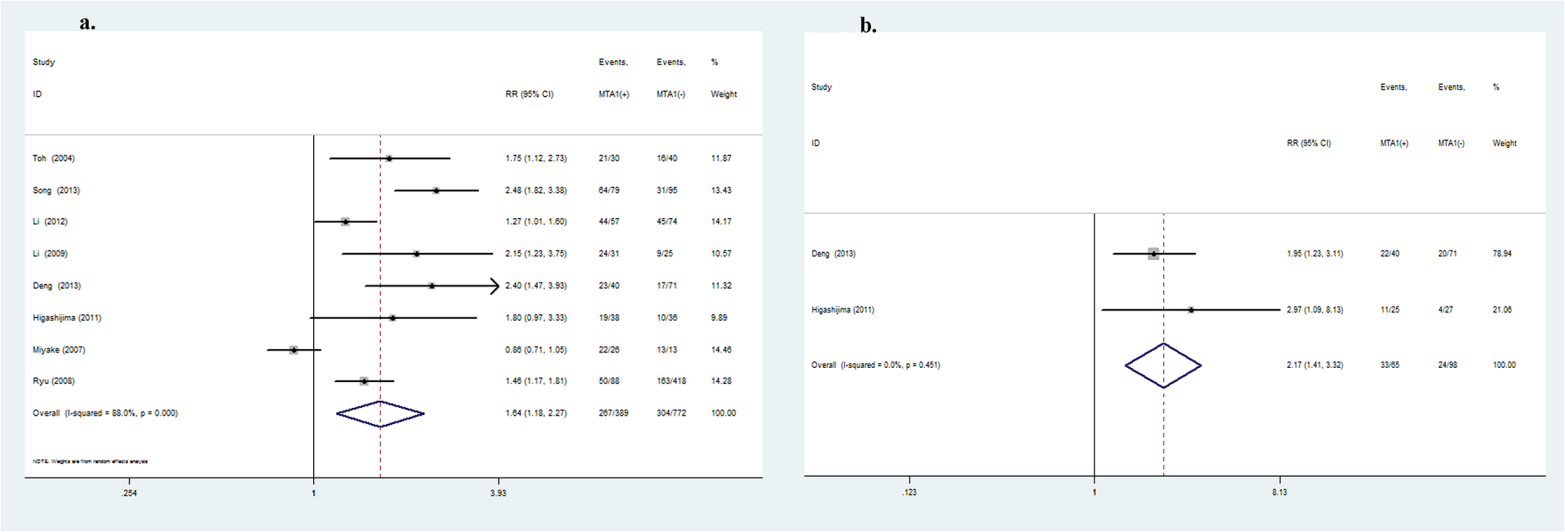
Forrest plot of the risk ratio for the association of MTA1 and 5-year OS/DFS in DTC patients. (4a) Association between MTA1 overexpression and 5-year OS. (4b) Association between MTA1 overexpression and 5-year DFS.

**Table 4:** Meta-analysis of a putative association between OS/DFS and MTA1 expression in digestive tract cancer.

OS/DFS	Number of studies	Number of patients	Heterogeneity		Model	RR(95%CI)	*P* value
			*I*^2^(%)	*P* value			
1-year OS	11	1722	57	0.009	RE	1.84(1.18,2.89)	**0.008**
3-year OS	11	1722	77	0	RE	1.74(1.32,2.30)	**<0.001**
5-year OS	8	1161	88	0	RE	1.64(1.18,2.27)	**0.003**
1-year DFS	3	224	16	0.31	FE	4.16(1.35,12.81)	**0.013**
3-year DFS	3	224	56	0.10	RE	1.90(1.02,3.53)	**0.044**
5-year DFS	2	163	0	0.45	FE	2.17(1.41,3.32)	**<0.001**

OS:overall survival; RR: risk ratio; CI: confidence interval; FE: fixed-effect model; RE: random-effect model

### Subgroup analyses

In order to further investigate the relationship between MTA1 and prognosis of DTC, all the eligible studies were divided into several subgroups according to the quality of each study and tumor type (Table 5). High-quality studies were divided into high quality studies subgroup. And according to tumor type, we investigated MTA1 expression in patients with gastrointestinal cancers (GI cancers) or EC.

**Table 5:** Subgroup analysis: Meta-analysis of the association between clinicopathological parameters and MTA1 expression.

Subgroup type	Parameters	Number of studies	Number of patients	Heterogeneity		Model	OR(95%CI)	*P* value
				*I*^2^(%)	*P* value			
High quality studies	Sex (male/female)	8	1093	32	0.17	FE	0.77(0.59,1.02)	0.72
	Age (<60/>60)	2	278	0	0.70	FE	0.96(0.59,1.56)	0.87
	Tumor size (<5cm/>5cm)	5	765	54	0.07	RE	0.75(0.47,1.22)	0.25
	Differentiation (well/poor)	6	681	54	0.05	RE	1.06(0.63,1.78)	0.84
	Depth of invasion (T1+T2/T3+T4)	6	759	87	0	RE	1.83(0.77,4.33)	0.17
	LN metastasis (positive/negative)	6	700	0	0.72	FE	2.62(1.89,3.63)	**<0.001**
	Tumor stage (early/advanced)	4	343	22	0.28	FE	3.81(2.38,6.12)	**<0.001**
	Vascular invasion(positive/negative)	3	533	0	0.78	FE	1.95(1.31,2.90)	**0.001**
Gastrointestinal cancers	Sex (male/female)	10	1149	0	0.51	FE	0.96(0.73,1.25)	0.75
	Age (<60/>60)	2	278	0	0.70	FE	0.96(0.59,1.56)	0.87
	Tumor size (<5cm/>5cm)	6	747	65	0.01	RE	0.49(0.28,0.87)	**0.01**
	Differentiation (well/poor)	8	929	42	0.10	FE	1.02(0.75,1.39)	0.88
	Depth of invasion (T1+T2/T3+T4)	9	1118	79	0	RE	1.88(1.05,3.37)	0.03
	LN metastasis (positive/negative)	8	948	0	0.71	FE	2.33(1.77,3.06)	**<0.001**
	Metastasis (positive/negative)	1	74	–	–	–	1.30(0.50,3.37)	0.58
	Tumor stage (early/advanced)	6	591	52	0.06	RE	2.78(1.63,4.74)	**<0.001**
	Vascular invasion(positive/negative)	3	304	0	0.86	FE	2.22(1.38,3.56)	**0.001**
Esophageal cancer	Sex (male/female)	5	662	0	0.62	FE	0.95(0.67,1.36)	0.80
	Age (<60/>60)	1	197	–	–	–	0.91(0.51,1.60)	0.73
	Tumor size (<5cm/>5cm)	3	395	0	0.69	FE	0.66(0.43,1.00)	**0.05**
	Differentiation (well/poor)	5	662	0	0.44	FE	0.82(0.57,1.18)	0.29
	Depth of invasion (T1+T2/T3+T4)	5	662	0	0.49	FE	2.60(1.88,3.61)	**<0.001**
	LN metastasis (positive/negative)	4	572	0	0.65	FE	2.17(1.55,3.04)	**<0.001**
	Tumor stage (early/advanced)	3	375	0	0.45	FE	2.54(1.66,3.88)	**<0.001**
	Vascular invasion(positive/negative)	1	70	–	–	–	2.64(0.97,7.14)	0.06
Gastric cancer	Sex (male/female)	3	332	0	0.89	FE	0.68(0.41,1.12)	0.13
	Tumor size (<5cm/>5cm)	2	271	0.92	0	RE	0.29(0.03,3.14)	0.32
	Differentiation (well/poor)	1	111	–	–	–	2.08(0.95,4.57)	0.07
	Depth of invasion (T1+T2/T3+T4)	2	271	18	0.27	FE	2.01(1.14,3.54)	**0.02**
	LN metastasis (positive/negative)	2	221	0	0.50	FE	3.35(1.72,6.51)	**<0.001**
	Tumor stage (early/advanced)	1	61	–	–	–	14.29(3.49,58.54)	**<0.001**
	Vascular invasion(positive/negative)	1	160	–	–	–	1.96(1.02,3.77)	**0.045**
Colorectal cancer	Sex (male/female)	2	155	0	0.64	FE	1.80(0.91,3.54)	0.09
	Age (<60/>60)	1	81	–	–	–	1.13(0.44,2.92)	0.81
	Tumor size (<5cm/>5cm)	1	81	–	–	–	0.37(0.14,0.98)	**0.045**
	Differentiation (well/poor)	2	155	63	0.10	FE	1.58(0.65,3.86)	0.32
	Depth of invasion (T1+T2/T3+T4)	2	155	94	0	RE	0.66(0.05,8.21)	0.74
	LN metastasis (positive/negative)	2	155	0	0.32	FE	2.11(1.08,4.13)	**0.03**
	Metastasis (positive/negative)	1	74	–	–	–	1.30(0.50,3.37)	0.58
	Tumor stage (early/advanced)	2	155	53	0.15	FE	1.81(0.91,3.58)	0.09
	Vascular invasion(positive/negative)	1	74	–	–	–	2.47(0.96,6.34)	0.06

LN metastasis: lymph node metastasis; OR: odds ratio; CI: confidence interval; FE: fixed-effect model; RE: random-effect model

TNM stages are based on tumor-node-metastasis classification advocated by International Union against Cancer

**Fig 5:**
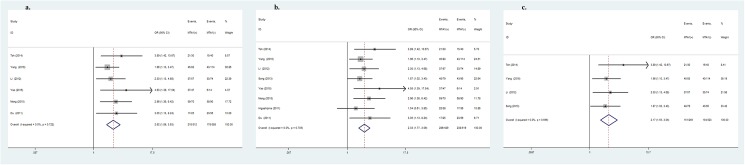
Subgroup analysis:Forrest plot of odds ratio for the association of MTA1 overexpression and lymph node metastasis (5a) in high-quality studies. Forrest plot of odds ratio for the association of MTA1 overexpression and lymph node metastasis (5b) in gastrointestinal cancers. Forrest plot of odds ratio for the association of MTA1 overexpression and lymph node metastasis (5c) in esophageal cancer.

In the subgroup containing high-quality studies, similar results of MTA1 expression showed a higher risk of lymph node metastasis (OR = 2.62, 95%CI: 1.89–3.63, *P*<0.001, Fig 5A), advanced TNM stage of DTC (OR = 3.81, 95%CI: 2.38–6.12, *P*<0.001) and a greater possibility of vascular invasion (OR = 1.95, 95%CI: 1.31–2.90, *P* = 0.001). However, MTA1 expression was not related to any other clinical parameters. All studies were of high quality, and the results were reliable.

MTA1 is also associated with metastasis-related clinical variables and prognosis in patients with GI cancers (including EC, GC and CRC). MTA1 is inextricably associated with depth of invasion (OR = 1.88, 95%CI: 1.05–3.37, *P* = 0.03), lymph node metastasis (OR = 2.33, 95%CI: 1.77–3.06, *P*<0.001, Fig 5B), TNM stage (OR = 2.78, 95%CI: 1.63–4.74, *P*<0.001) and vascular invasion (OR = 2.22, 95%CI: 1.38–3.56, *P*<0.001) of GI cancers. MTA1 was significantly related to EC in GI cancers. Elevated expression of MTA1 was always associated with depth of invasion (OR = 2.60, 95%CI: 1.88–3.61, *P*<0.001), lymph node metastasis (OR = 2.17, 95%CI: 1.55–3.04, *P*<0.001, Fig 5C), and TNM stage (OR = 2.54, 95%CI: 1.66–3.88, *P*<0.001), consistent with previous meta-analysis. Moreover, MTA1 high expression is relatively association with the clinicopathological variables of GC and CRC patients (Table 5).

**Fig 6:**
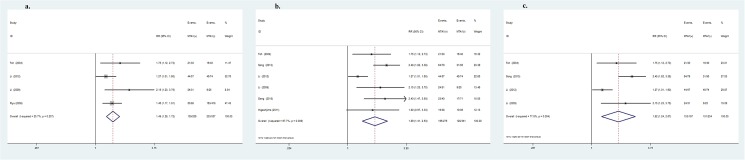
Forrest plot of the risk ratio for the association of MTA1 and OS in DTC patients:Association between MTA1 overexpression and 5-year (6a) OS in high-quality studies subgroup. Association between MTA1 overexpression and 5-year (6b) OS in gastrointestinal cancer subgroup. Association between MTA1 overexpression and 5-year (6c) OS in esophageal cancer subgroup

In all the subgroups (Table 6), MTA1-positive expression was strongly correlated with 1-, 3- and 5-year OS. Among the high-quality studies, MTA1 expression was associated with 1- (RR = 1.96, 95%CI: 1.11–3.44, *P* = 0.02), 3- (RR = 1.73, 95%CI: 1.20–2.49, *P* = 0.003) and 5- (RR = 1.49, 95%CI: 1.28–1.72, *P*<0.001, Fig 6A) year OS. Further, GI patients with increased MTA1 expression manifest shorter 1- (RR = 1.66, 95%CI: 1.21–2.26, *P* = 0.001), 3- (RR = 1.87, 95%CI: 1.27–2.26, *P* = 0.002) and 5- (RR = 1.89, 95%CI: 1.41–2.53, *P*<0.001, Fig 6B) year OS. Similar to GI cancers, MTA1-positive expression increased the risk of death postoperatively. MTA1 was linked to 1- (RR = 1.39, 95%CI: 1.01–1.91, *P* = 0.04), 3- (RR = 1.75, 95%CI: 1.06–2.88, *P* = 0.03) and 5- (RR = 1.82, 95%CI: 1.24–2.67, *P* = 0.002, Fig 6C) in EC patients.

**Table 6:** Subgroup analysis: Meta-analysis of the association between OS and MTA1 expression.

Subgroup type	OS	Number of studies	Number of patients	Heterogeneity		Model	RR(95%CI)	*P* value
				*I*^2^(%)	*P* value			
High quality studies	1-year OS	7	1324	65	0.01	RE	1.96(1.11,3.44)	**0.02**
	3-year OS	7	1324	81	0	RE	1.73(1.20,2.49)	**0.003**
	5-year OS	4	763	26	0.26	FE	1.49(1.28,1.72)	**<0.001**
Gastrointestinal cancers	1-year OS	8	874	48	0.06	FE	1.66(1.21,2.26)	**0.001**
	3-year OS	8	874	80	0	RE	1.87(1.27,2.75)	**0.002**
	5-year OS	6	616	68	0.01	RE	1.89(1.41,2.53)	**<0.001**
Esophageal cancer	1-year OS	5	689	48	0.10	FE	1.39(1.01,1.91)	**0.04**
	3-year OS	5	689	85	0	RE	1.75(1.06,2.88)	**0.03**
	5-year OS	4	431	78	0.004	RE	1.82(1.24,2.67)	**0.002**
Gastric cancer	1-year OS	2	172	0	0.85	FE	7.03(1.32,34.47)	**0.02**
	3-year OS	2	172	79	0.03	RE	2.21(0.80,6.10)	0.13
	5-year OS	1	111	–	–	–	2.40(1.47,3.93)	**<0.001**
Colorectal cancer	1-year OS	1	74	–	–	–	2.37(0.49,11.44)	0.28
	3-year OS	1	74	–	–	–	2.17(1.01,4.64)	**0.047**
	5-year OS	1	74	–	–	–	1.80(0.97,3.33)	0.06

OS:overall survival; RR: risk ratio; CI: confidence interval; FE: fixed-effect model; RE: random-effect model

### Sensitivity analysis and publication bias

In order to test the robustness of RR estimates in OS, sensitivity analysis was conducted by individually excluding studies and analyzing the effects of the remaining studies. Sensitivity analysis (S1 Fig) indicated that the RR estimates were relatively reliable and credible as no point estimate of the omitted study fell outside the 95% CI.

**Fig 7:**
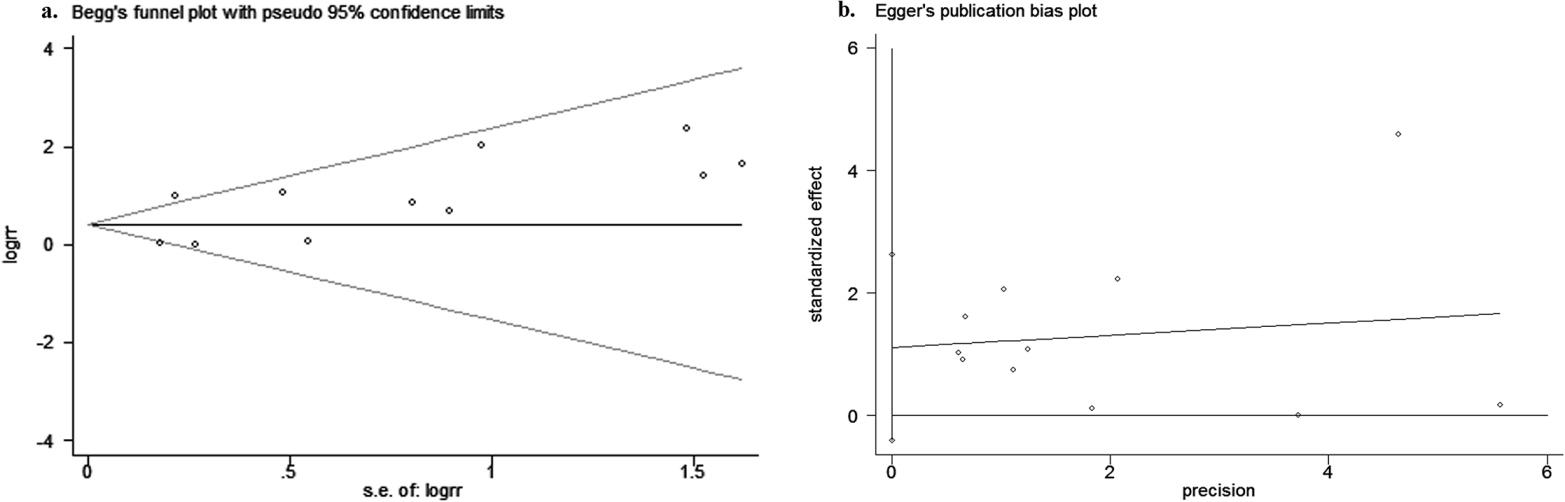
Begg’s funnel plot (7a) (*P* = 0.35) and Egger’s funnel plot (7b) (*P* = 0.13) for possible publication bias test of this study. There was no publication bias and the results are credible

Begg's rank correlation and Egger's weighted regression methods were used to statistically assess publication bias. As shown in Fig 7A and 7B, neither Begg’s (*P* = 0.35) nor Egger’s (*P* = 0.13) test provided a clear evidence of publication bias. No publication bias was detected in the current study. The results reported in this article are credible.

## Discussion

Depth of invasion (T), lymph node metastasis (N), and the presence of distant metastasis (M)—TNM stage was considered as the most important prognostic factors for DTC, such as GC [21]. However, large clinical case studies suggest that patients at similar pathological stages of DTC may differ substantially in survival after complete surgical resection. Therefore, the current staging system is inadequate for accurate prognosis. Prognosis of DTC is always predicted by TNM staging clinically. However, TNM staging lacks sensitivity. In our opinion, EC and liver cancer always show a high risk of tumor recurrence and metastasis, despite complete resection or targeted therapy. Several deaths among DTC patients are still attributed to local recurrence and/or distant metastasis. A new prognostic marker is needed to identify patients with poor survival time or indicate those with a higher risk of tumor metastasis.

Members of the MTA family play a vital role in both physiological and pathophysiological processes, especially in cancer development and distant metastasis. MTA family members regulate metastasis. MTAs including MTA1, MTA2, and MTA3 are expressed indifferent isoforms (MTA1,MTA1s,MTA-ZG29p,MTA2,MTA3, and MTA3L) [22–23]. MTA1 is a founding member of this family and was first identified as a metastasis-associated tumor gene differentially expressed in rat metastatic tumors [24]. MTA1 overexpression has been identified in many cancers. However, the molecular functions of MTA1 were unclear until it was identified as an integral component of the NuRD complex [25]. Luo *et al*. [26] conducted a meta-analysis to further investigate the role of MTA1 in solid tumors, and confirmed that MTA1 expression was significantly associated with prognosis of solid cancers. Currently, the clinical and prognostic value of MTA1 in DTC is unknown. Ning *et al*. [27] reviewed the expression and clinical significance of MTA family, and concluded that MTA1 expression was correlated with invasion and lymph node metastasis in GI cancer. However, the prognostic value of MTA1 expression in DTC is unclear and controversial. Several studies found that MTA1-positive expression was not correlated with OS in patients with esophageal squamous cell carcinoma and breast cancer [10, 28].

In our study, we investigated the overexpression of MTA1 and clinicopathological parameters in DTC. The results demonstrate that MTA1-positive expression increased the risk of stomach wall invasion (OR = 1.88, 95%CI: 1.05–3.37, *P* = 0.03), lymph node-positive metastasis (OR = 2.30, 95%CI: 1.76–3.01, *P*<0.001) and vascular invasion (OR = 2.02, 95%CI: 1.40–2.91, *P*<0.001), leading to later TNM stages (OR = 2.78, 95%CI: 1.63–4.74, *P*<0.001). Furthermore, MTA1 expression was not only linked to OS, but also showed significant association with DFS. DTC patients with MTA1-positive expression always manifested shorter OS and DFS. Similar conclusions were obtained in the three different subgroups. MTA1 expression was tightly associated with clinicopathological parameters and 1-, 3-, 5-year OS in GI cancer and EC.

From a clinical perspective, MTA1 over-expression was strongly and independently correlated with depth of invasion, lymph node metastasis, vascular invasion and TNM stage. Tumor tissues expressing MTA1 show deeper invasion into the lymphatic network under the mucosa. Vascular invasion leads to advanced tumor stages, and shortens the OS of patients with DTC. Previous studies suggested that the MTA1 gene acted as a transcriptional regulator, in conjunction with other components of NURD to mediate transcriptional repression and the association of repressor molecules with chromatin [23,29–30]. For example, MTA1 protein physically interacts with HDAC1 [31]. The two proteins are the key components of NuRD complex, which contains histone deacetylase. Histonedeacetylation alters chromatin structure and transcriptional control. Toh *et al*. [9] observed that MTA1 expression in ESCC was associated with the activity of H4 histone deacetylase. Tumor suppressor genes including p53, p21 and Bcl-2 are regulated by histone acetylation [32–33].

The limitations of this meta-analysis are as follows: (1) A few eligible non-English publications were excluded; (2) IHC assessments of MTA1 were still discordant; and (3) The number of articles was inadequate. Nonetheless, the meta-analysis has several advantages: (1) This study is the first of its kind to investigate the association between MTA1 overexpression and clinicopathological parameters in DTC; (2) The study successfully evaluated the association of MTA1 expression with the OS/DFS of DTC patients.

This work was supported by grants from the National Natural Science Foundation of China (NO: 81602425) and the Natural Science Foundation of Anhui Province (NO: 1508085QH152,1608085MH163). The funders had no role in study design, data collection and analysis, manuscript preparation, or submission for publication.

In conclusion, MTA1 expression is significantly associated with clinicopathological parameters, DFS and OS in DTC patients. It may play an independent role in predicting aggressive tumor behavior and poor prognosis. The results of the meta-analysis suggest that MTA1 is a potential target for anticancer therapy. Further investigations are needed to identify the mechanisms underlying the role of MTA1.

## Supporting information

S1 Checklist The checklist of this meta-analysis. (DOC)

S1 Fig Sensitivity analysis: It indicated that eligible articles were relatively reliable and credible. (TIF)
