# Supplementary figures and images for: Role of metastasis-associated protein 1 in prognosis of patients with digestive tract cancers: A meta-analysis
Source: PLoS One. 2017 Jun 1;12(6):e0176431. doi: 10.1371/journal.pone.0176431 (PMC5453427; doi:10.1371/journal.pone.0176431)

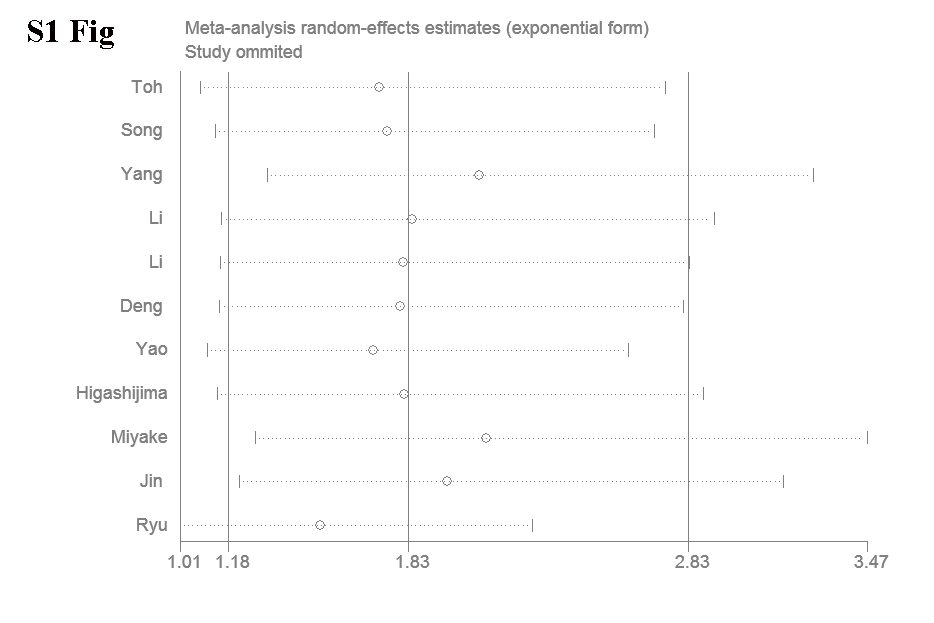

Supplement: S1 Fig — (TIF) [file pone.0176431.s002.tif]
